# Psychodynamic profiles of major depressive disorder and generalized anxiety disorder in China

**DOI:** 10.3389/fpsyt.2024.1312980

**Published:** 2024-01-23

**Authors:** Jia Xu, Yuxi Wang, Yujia Peng

**Affiliations:** ^1^ Peking University Institute of Mental Health, Key Laboratory of Ministry of Health (Peking University), Beijing, China; ^2^ School of Psychological and Cognitive Sciences and Beijing Key Laboratory of Behavior and Mental Health, Peking University, Beijing, China; ^3^ Institute for Artificial Intelligence, Peking University, Beijing, China; ^4^ National Key Laboratory of General Artificial Intelligence, Beijing Institute for General Aritificial Intelligence, Beijing, China

**Keywords:** operationalized psychodynamic diagnosis, major depressive disorder (MDD), generalized anxiety disorder (GAD), psychodynamic profile, personality organization

## Abstract

Traditional clinical diagnoses relying on symptoms may overlook latent factors that illuminate mechanisms and potentially guide treatment. The Operationalized Psychodynamic Diagnosis (OPD) system may compensate for symptom-based diagnosis by measuring psychodynamic profiles underlying mental disorders through conflicts and structure axes. However, OPD has not been widely adopted in China, and it remains unclear whether OPD can be used as an effective approach to distinguish between depression and anxiety. The current study aims to adopt the OPD system to investigate the psychodynamic profiles of major depressive disorder (MDD) and generalized anxiety disorder (GAD) in China, targeting patients with “pure” symptoms without comorbidity. We recruited 42 MDD patients, 32 GAD patients, and 31 healthy controls (HC), and assessed their self-report depression and anxiety symptoms, along with their underlying psychodynamic profiles through OPD interviews. Overall, both MDD and GAD patients showed more prominent conflict issues and lower levels of structure than HC. The MDD and GAD groups yielded different conflict profiles and conflict processing modes when processing their second conflicts. Importantly, the multi-dimensional psychodynamic profiles achieved machine learning classification of clinical groups with an accuracy of 0.84, supporting successful distinction of MDD and GAD patients. In conclusion, the OPD demonstrated sensitivity in revealing distinct psychodynamic profiles underlying “pure” depression and anxiety clinical populations in China. This work calls for future incorporation of OPD as a tool to investigate psychodynamic formulations underlying mental disorders, compensating for traditional symptom-based diagnostic approaches to guide precise individualized interventions.

## 1 Introduction

Major depressive disorder (MDD) and generalized anxiety disorder (GAD) are the top two mental diseases worldwide, severely impacting patients’ psychosocial functioning and quality of life (1, 2). In China, there is a comparable high prevalence of MDD and GAD, with 12-month prevalence estimates of 2.3% and 0.8% and lifetime prevalence estimates of 3.3% and 1.2%, respectively (3, 4). Currently, diagnoses of these mental disorders are based on symptomatology, which results in poor separation of clinical symptom profiles, leading to heterogeneity and comorbidity. This further complicates the identification of appropriate therapeutic interventions and increases the challenges of treatment (5).

To disentangle the intertwined symptoms, psychodynamics provides a unique perspective to investigate the hidden psychological mechanisms in MDD and GAD (6). The Operationalized Psychodynamic Diagnosis (OPD) system (7) is a clinical tool that can provide a multiaxial psychodynamic description for mental illnesses, containing in its second revision (OPD-2) five independent axes: (I) Experience of illness and prerequisites for treatment, (II) Interpersonal relations, (III) Conflict, (IV) Structure, and (V) Mental and psychosomatic disorders. Specifically, Axes III “Conflicts” and IV “Structures” are commonly the only axes being focused on when investigating the psychodynamic profiles of patients, as they align to the psychodynamic core concepts: conflict and structure (8), while interpersonal relations are not rated dimensionally, making the scientific use of OPD axis II difficult and the other two axes play as supplemental assessment from the perspectives outside the psychodynamics. Consistently, Benecke et al. also specified the conflict and structure axes of OPD to explain individual psychodynamic differences (9). Psychodynamic conflicts correspond to the internal and unconscious collision and tensions of opposed tendencies of motives, needs, and wishes. The conflict axis in OPD focuses on the contents and coping style of conflicts, indicating patients handle the following conflicts in a passive or active way: individuation (dependence vs. autonomy), control (submission vs. control), care (need for care vs. autarky), self-worth (undervaluing oneself vs. overvaluing oneself), guilt (self-blame vs. rejecting responsibility), and sexual roles (unattractiveness vs. attractiveness) and identity (lacking identities vs. adoption of identity) (10). Axis IV comprises four main parts: “perception”, “regulation”, “emotion communication” and “attachment”, where each part is rated on a self-related and an object-related dimension, describing the relationships and interaction with self and objects. Together, the OPD structure axis resembles the personality functioning concept as defined in the Alternative DSM-5 Model for Personality Disorders and ICD-11.

Together, the OPD system incorporates perspectives of psychoanalysts, psychosomatic medicine, and psychiatrists in psychotherapy research. The OPD system has become a standard tool for psychotherapy assessment in Germany and has been published and used in several research in English, Portuguese or Spanish (11, 12). Chinese clinical practitioners introduced and adapted this tool in 2009 (13), but it has not been applied in the local clinical settings due to uncertainties in distinguishing the latent psychodynamics of psychiatric illnesses and a lack of comprehensive investigations on the psychodynamic formulations of patient populations under Chinese culture.

In the previous empirical research, MDD and GAD were found to be both associated with abnormalities in low self-esteem, insecure attachment, immature defense mechanisms, common abnormal personality (such as neuroticism and introversion/extraversion), and the processing and regulation of emotion (14–22) corresponding to issues on each indicator in Axis IV structures. Along Axis III in the psychodynamic framework, MDD may be rooted in patients’ unresolved conflicts with others sharing close relationships, giving rise to unconscious feelings of anger and guilt directed towards themselves or others. MDD patients may struggle with these negative feelings due to an immature defense mechanism. Meanwhile, GAD can be attributed to the patient’s excessive worry and anxiety, potentially stemming from an overactive defense mechanism against unconscious fears and perceived threats. The threat may be rooted from insecure/conflicted attachment patterns (23), leading to deficits in self-structure and abnormal defense mechanisms. For instance, the immature defense styles “pseudo altruism” and “reaction formation” were frequently identified in Chinese GAD patients (24).

Axis IV structure level can offer valuable information to predict symptom severity and effectiveness of treatment, thereby guiding the treatment plan. Studies have shown that the greater structural vulnerability of emotional self-regulation influences the severity of depressive symptoms (25). Moreover, the structural level of personality function described in the OPD Structure Questionnaire (OPD-SQ) was found to be associated with the quality of life of depressive patients (26). Will and colleagues (27) found that better structural integration is associated with great capability of reflection, impulse control, and frustration tolerance in depressed patients. For patients with lower levels of structural integration or deficits, supportive therapy including psychoeducational interventions and advice may be necessary in therapy (8). Therefore, these patients may benefit from different intervention methods, shifting back and forth along the expressive-supportive continuum based on their conflict/structure profiles and psychotherapy phrases (8, 28). The expressive-supportive continuum intervention categories include interpretation, observation, confrontation, clarification, encouragement to elaborate, empathic validation, psychoeducational interventions, and advice and praise.

Although psychodynamic profiles have been widely investigated and reported under the western culture, we cannot directly adopt those conclusions to guide our clinical settings due to the impact of cultural factors on clinical presentations. The World Health Organization stated that cross-cultural applicability was a very high priority during the process of revising the symptom-based clinical diagnosis system (29), indicating the existence of culture-specific symptoms. This phenomenon was observed in the variation of the core symptoms of depression and anxiety. Rumination was found to be the core symptom in western culture, while worry instead of rumination plays a central role in the eastern culture (30, 31). Chinese GAD patients were found to use the defense style “pseudo altruism”, and “reaction formation” significantly more than other cultures, manifesting as coping more with repressed rage (24). In addition, the variations in clinical presentations also indicate the possible difference in psychodynamic formulations. Based on OPD, German patients suffered from more frequent conflicts including “need for care vs. autarky” and “guilt conflict”, while Chinese patients had more frequent “dependence vs. individuation” conflicts (32). Although Xu & Cierpka’s study provides insights into the cultural variations in the Conflicts axis, it lacks the psychodynamic profiles of two groups and specific clinical features of the two groups. Therefore, to facilitate the application of the OPD system in MDD and GAD clinical diagnosis and treatment planning, it is necessary to investigate the comprehensive psychodynamic profiles of these two groups in Chinese culture, and a comparison with western findings can help refine the treatments.

In the current study, we aim to examine the sensitivity of Axis III and IV in the OPD system and explore the psychodynamic profiles of MDD and GAD patients in China. Aiming to reveal psychodynamic profiles specifically for each mental disorder, we only recruited patients with a single or “pure” diagnosis, avoiding comorbidity conditions. In the current study, we hypothesize that data will yield similar conflict profiles, such as conflicts in self-worth and guilt, in both MDD and GAD. Additionally, we hypothesize that the strategies for coping with conflicts will be different between clinical groups based on existing research evidence, where MDD individuals may employ more passive coping style (32, 33), and GAD individuals may adopt more active coping style such as “reaction formation” and “pseudo altruism” (24, 33). For the structure levels, we hypothesize that both MDD and GAD patients will demonstrate lower levels of structural integration compared to healthy controls. We further expect MDD patients to exhibit the lowest levels of structural integration due to their utilization of passive conflict processing models, which are more associated with personality functioning impairments (10) in contrast to those with GAD.

## 2 Method

### 2.1 Participants

The total sample contains 42 MDD patients (29 female, mean age = 35.05 years, *SD* = 11.00) including 7 inpatients, 32 GAD outpatients (21 female, mean age = 39.97 years, *SD* = 11.12), and 31 healthy controls (20 female, mean age = 37.87 years, *SD* = 10.22). Both MDD and GAD patients were diagnosed by experienced psychiatrists based on the DSM-IV-TR criteria (American Psychiatric Association, 2000), and they were recruited in two independent research projects and in a multi-sited way. They had no other comorbid psychiatric or organic illness that could impact cognitive functions. Healthy controls were excluded if they had any history of psychiatric or neurological disorders, history, or current use of any psychoactive medication. The three groups are matched in marriage status, educational attainment, and occupation status, except for patient care (**Table 1**). This study was approved by the ethics committee of Peking University Sixth Hospital (Institute of Mental Health) in China, and all participants gave their informed written consent in accordance with the Declaration of Helsinki.

**Table 1:** Demographic characteristics of the participants.

Variables		MDD group (n=42)	GAD group (n=32)	Control group (n=31))	H/F/χ^2^	p
Age (years) (M ± SD)		35.05 ± 11.00	39.97 ± 11.12	37.87 ± 10.22	1.927	0.151
Gender N (%)	Male	13(31.0)	11(34.4)	11(35.5)	0.186	0.911
	Female	29(69.0)	21(65.6)	20(64.5)		
Marital status N (%)	single	10(23.8)	5(15.6)	9(29.0)	1.261	0.532
	married	24(57.1)	24(75.0)	19(61.3)		
	divorce	6(14.3)	3(9.4)	3(9.7)		
	widowed	2(4.8)	0	0		
Educational attainment N (%)	Lower than undergraduate	27(64.3)	22(68.8)	15(48.4)	0.906	0.636
	Undergraduate or higher	15(35.7)	10(31.2)	16(51.6)		
Occupation status N (%)	retired	5(11.9)	7(21.9)	2(6.5)	0.818	0.664
	employed	30(71.4)	20(62.5)	27(87.1)		
	unemployed	7(16.7)	5(15.6)	2(6.5)		
Age of onset (M ± SD)		31.62 ± 11.72	35.66 ± 12.39	–	-1.43	0.156
Patient care N (%)	inpatient	7(16.7)	0(0)	–	11.250	0.004**
	outpatient	35(83.3)	32(100)	–		

* p < 0.05 ** p < 0.01 *** p < 0.001. MDD, major depressive disorder; GAD, generalized anxiety disorder; M, mean; SD, standard deviation; N, number.

### 2.2 Clinical assessments

#### 2.2.1 Hamilton depression rating scale

Depression severity was measured by HAMD-17 (The 17-item Hamilton Depression Rating Scale, Hamilton, 1967), which is a widely used clinician-administered depression assessment tool utilizing a scale of 0 (not present) to 4 (severe). It was developed by Hamilton, where high scores indicate more serious depressive symptoms. The Chinese version has high inter-rater reliability with r = 0.88–0.99 (34).

#### 2.2.2 Hamilton anxiety rating scale

The severity of anxiety symptoms was clinician-rated by a 14-item Hamilton Anxiety Rating Scale (HAMA, Hamilton, 1959). Each item is scored on a scale of 0 (not present) to 4 (severe), with a total score range of 0–56, where < 17 indicates mild severity, 18–24 mild to moderate severity, and 25–30 moderate to severe. The HAMA has high reliability in China with r = 0.93 (35).

#### 2.2.3 Operationalized psychodynamic diagnosis interview

Psychodynamic diagnosis of conflicts (Axis III) and structures (Axis IV) were clinically assessed by two experienced clinicians using the Chinese OPD interview, which was introduced and translated in 2009 (13). In Jiang’s study, the interrater reliabilities for the conflict and structure axes were acceptable, with values of 0.65 and 0.51, respectively, and the 3–5 weeks retest reliabilities were high, with values of 0.81 for the conflict axis and 0.93 for the structure axis. The inter-rater reliability was also verified through prior practice by the same team, and a good agreement between the two raters was found for the conflict axis, with a value of 0.81, and a moderate agreement was found for the structure axis, with a value of 0.58. These results indicate that the psychodynamic diagnosis of conflicts and structures using the Chinese OPD interview in the current study is reliable and valid for use in clinical practice and research.

### 2.3 Statistical analysis

Statistical analysis was performed by IBM Statistical Package for the Social Sciences (SPSS V20.0). To examine the characteristics of different groups, demographic data, HAMD and HAMA scores, and OPD dimensions among the three groups were compared respectively, by applying one-way ANOVAs and repeated measures ANOVAs for continuous variables and Kruskal-Wallis test or chi-square test for categorical variables, the effect of patient care (inpatient vs. outpatient) was controlled as a covariate.

To further evaluate whether we can use psychodynamic profiles to guide clinical prediction and classification, we utilized the Least Absolute Shrinkage Selection Operator (LASSO) regression, using the *glmnet* package (36)in R. We used all 20 OPD indicators as predictive variables (see **Supplementary Table 2**), and groups (MDD, GAD, or HC) as the outcome variable. Data were split into 80% vs. 20% for training and testing respectively. Nested cross-validation with 5 folders was employed during training. The sampling, training, and testing procedures were repeated 100 times as a bootstrapping strategy to examine the confidence interval of the predictive accuracy. Furthermore, to examine the effectiveness of OPD Axes III and IV in group classification, we summarized the frequency of each feature being selected as an effective predictor across 100 iterations of model fitting.

## 3 Results

### 3.1 Demographic information

The effectiveness of the grouping was examined and found to be reasonable (**Table 1**), as there were significant differences among groups in both HAMD (*F*(2, 102)=94.402, *p*<0.001) and HAMA (*F*(2, 98)=114.712, *p*<0.001). The *post-hoc* test revealed that MDD (*M±SD* = 17.67±6.80) group had significantly higher HAMD scores than those in both the GAD (*M±SD* = 14.06±4.67, *p*=0.026) and HC (*M±SD*=1.45±1.72, *p*<0.001) groups, and individuals in the GAD group (*M±SD*=23.46±7.45) exhibited the most severe anxiety symptoms as assessed by HAMA when compared to those in the MDD (*M ± SD*=12.05 ± 5.57, *p*<0.001) and HC groups (*M±SD*=2.10±1.83, *p <*0.001). Both patient groups yielded significantly greater scores on HAMD and HAMA as compared to HCs (*p*s<0.001).

### 3.2 Psychodynamic profiles across groups

**Figure 1:**
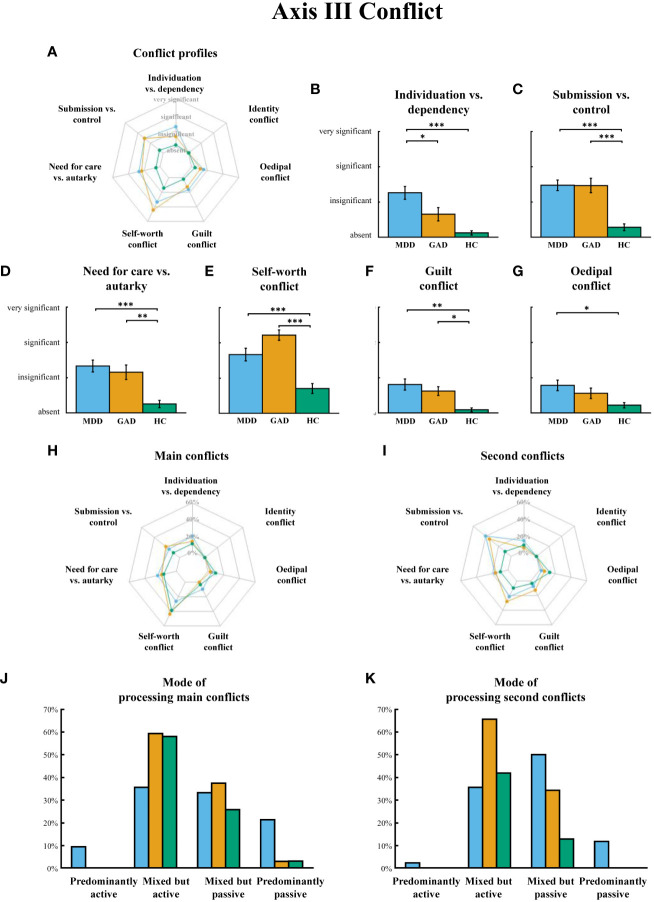
**(A)** OPD Axis III “conflict” profiles in MDD, GAD and H. **(B–G)** Significant group differences on conflict dimensions. **(H)** The composition of main conflict in three groups, expressed as the percentage of the group population. **(I)** The composition of second conflict in three groups, expressed as the percentage of the group population. **(J, K)** Their mode of processing main conflicts and second conflicts. MDD = major depressive disorder, GAD = generalized anxiety disorder, HC = healthy controls. Error bars indicate standard errors of the average across participants. * p < 0.05, ** p < 0.01, *** p < 0.001.

*Axis III Conflicts.* After controlling the effect of patient care (inpatient vs. outpatient), the repeated measures ANOVA determined that Axis III “conflict” significantly differed both among groups, *F*(2, 101)=145.709, *p*<0.001, *η^2^ =*0.743, and among conflict indicators (*F*(6, 606)=29.802, *p*<0.001, *η^2 ^=*0.228) (**Figure 1A**). The interaction effect between conflict indicators and groups was also significant (*F*(6, 606)=4.546, *p* < 0.001, *η^2^ =*0.083). However, no significant main of patient care or its interactive effects with other variables has been found (*ps*>0.05). One-way ANOVAs performed on each “conflict” indicator revealed significant group differences among three groups on the first six indicators (*ps*<0.015). Specifically, *post hoc* analysis revealed that individuals in the MDD group demonstrated stronger conflicts than HC on dimensions of “individuation vs. dependence”, “submission vs. control”, “need for care vs. autarky”, and “self-worth conflict”, “guilt conflict” and “oedipal conflict” (*ps*<0.010, **Figures 1B-G**). Comparing MDD and GAD, the MDD group yielded stronger conflicts on “individuation vs. dependence” (*p*=0.025, **Figure 1B**) and milder “self-worth conflict” (*p*=0.043, **Figure 1E**). The GAD group exhibited stronger conflicts than HC on “submission vs. control”, “need for care vs. autarky”, “self-worth conflict” and “guilt conflict” (*ps*<0.022, **Figures 1C-F**).

It is worth noting that a large proportion of patients in both the MDD (26.2%) and GAD (43.8%) groups identified “self-worth conflict” as their main conflict, with “submission vs. control” being the second most common conflict dimension (MDD: 40.5%; GAD: 34.4%) (**Figures 1H, I**). However, the assessments of the main and second conflict were independent, i.e., one patient may have “self-worth” as the main conflict but may have a different conflict dimension as their second conflict. Furthermore, one-way ANOVAs found that three groups had no differences in processing mode to main conflicts (*F*(2,98)=1.578, *p*=0.212), while they had significant differences in processing mode to second conflicts (*F*(2,88)=5.560, *p*=0.005), with MDD group exhibited a “mixed but passive” processing mode, which is significantly different from both GAD (*p*=0.027) and HC (*p*=0.018), who tend to process conflicts in a “mixed but positive” way (**Figures 1J, K**).

**Figure 2:**
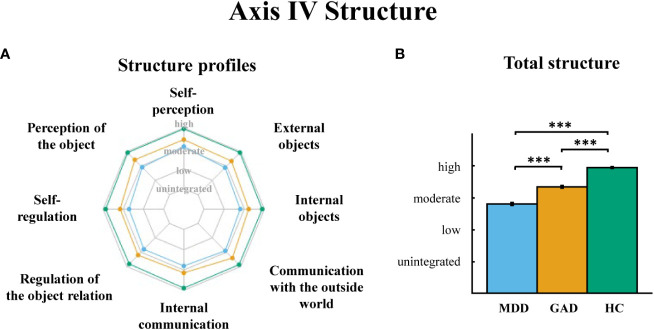
**(A)** OPD Axis IV “structure” profiles in MDD, GAD, and HC. **(B)** The total structure score comparisons among three groups. MDD = major depressive disorder, GAD = generalized anxiety disorder, HC = healthy controls. Error bars indicate standard errors of the average across participants. *** p < 0.001.

*Axis IV Structures*. Significant differences in the overall structure level among groups were found reflected by the total structure score (*F*(2,102)=202.512, *p*<0.001, **Figure 2B**). Consistently, after controlling the effect of patient care (inpatient vs. outpatient), we used a repeated measures ANOVA to examine the effect of group and structure indicators, with group as between-subject factor and structure indicators as within-subject factor. Results showed that the main effect of group was significant, *F*(2, 101)=209.164, *p*<0.001, *η^2^ =*0.806, as well as the main effect of structure indicators, *F*(7, 707)=13.523, *p*<0.001, *η^2^ =*0.118. The interaction effect between structure indicators and groups was also significant, *F*(14, 707)=2.278, *p*=0.005, *η^2^ =*0.043. Patient care did not show a significant main effect or interactive effect (*p*s>0.05). One-way ANOVAs on structure indicators further indicated the significant group differences (*p*s<0.001). *Post hoc* analysis revealed that both GAD (*p.s.* < 0.001) and MDD (*p*s<0.001) had overall lower structure level than HC, and MDD had the lowest structure levels (*p.s.* < 0.001) in all indicators (**Figure 2A**). All conflicts and structures profiles of groups were reported in See **Supplementary Table 1**.

### 3.3 Group classification based on psychodynamic profiles

The results of the LASSO regression analysis showed that the OPD Axis III and IV had high predictive and discrimination ability to classify three groups, with the predictive accuracy (*M* = 0.844, *CI* [0.650, 1.000]) and the mean area under the curve (AUC) was 0.920 *CI* [0.833, 1.000].

Looking at feature selections across iterations of model training (**Supplementary Table 2**), “self-worth conflict” and “total structure” were 100% and 99% selected as effective predictors in the LASSO regression model, followed by two structure indicators “self-regulation” and “attachment to internal objects” with frequencies of 96% and 98% respectively. It is important to note that although there was heterogeneity among the predictive OPD indicators in 100 model iterations, all iterations consistently suggested the OPD profiles can consistently predict and distinguish between clinical groups. For detailed statistical reports, see **Supplementary Table 2**.

## 4 Discussion

In the current study, we identified distinct psychodynamic profiles in MDD and GAD, manifesting as more prominent conflicts and lower levels of psychological structure as revealed by OPD Axes III and IV, compared to HC. These results serve as empirical support to the classic psychodynamic theories that MDD and GAD patients have more unconscious conflicts and intrapsychic structure deficits than healthy individuals (8, 26, 37, 38), extending previous evidence that distinguished the psychodynamic profiles between panic disorder patients and MDD patients (39), and studies that suggested different psychodynamic profiles between various anxiety disorders (37, 38).

For Axis III of conflicts, MDD and GAD groups yielded significantly different conflict profiles and conflict processing modes. First, the MDD group demonstrated stronger conflicts on “individuation vs. dependence”, whereas the GAD group yielded stronger conflicts on “self-worth conflict”. The results are consistent with previous evidence where lower self-esteem, insecure attachment and autonomy/independence conflict were found in MDD and GAD (6, 37, 40), and further provide evidence on the differences between MDD and GAD. Second, MDD patients tended to process major conflicts in a “mixed but passive” manner, while GAD patients tended to process them in a “mixed but positive” way when responding to their conflicts. By reviewing evidence-based unified psychodynamic protocol for depressive and anxiety disorders, Leichsenring and Steinert (6) found that different treatments may fit MDD and GAD better separately. Empowering patients to become active participants in the treatment may be specifically relevant for the treatment of depressive patients, while the inner dialogue may have a more calming tone and function for patients with GAD. The significantly different conflict models of MDD and GAD found in our study support these suggestions. Empowerment could alter the relatively passive conflict model in depression, while calming inner dialogue may reduce the active conflict model in anxiety.

Beyond Axis III of conflicts, MDD and GAD also demonstrated significant differences on Axis IV of structure. Specifically, both patient groups demonstrated lower psychological structure levels than the healthy controls, while individuals with MDD demonstrated all the lowest structural profiles. The finding of lower psychological structure in two clinical patient groups may reflect the group difference in immature defense mechanisms. Colovic et al. (41) found that depressive patients more frequently used immature defense mechanisms than anxiety patients. Therefore, MDD patients tend to have more deficits of intrapsychic structure than GAD patients. These lower-than-moderate structure level of MDD patients may be associated with the fact that the patients in the current study were purely diagnosed with MDD in this study, instead of other depressive disorders belonging to neurosis, such as dysthymia or depressive neurosis. MDD is more likely to bring worse symptoms than dysthymia or depressive neurosis. The lower the level of personality functioning or structure level, the higher the severity of depressive symptoms (26). Especially, MDD group and GAD group had significantly different patient care, where the MDD group included 7 inpatients, who often had worse symptoms than outpatients. The current results are also consistent with the previous studies showing structure levels of GAD between moderate and high, along with most clinical neuroses (7). The finding supports the idea that psychodynamic treatment for anxiety focuses on unconscious conflicts and the defense use (23, 42), such as expressive interventions (6). However, structure levels of GAD between moderate and high in the present study are different from findings revealed by Doering and colleagues (22), where they found GAD spanning across all levels of personality organization, from high to low level. Their patients were recruited from both inpatient and outpatient care settings. While the GAD patients in the present study were only in outpatient care settings. Patients from different mental health care settings would have found different personality functioning (22). It is possible GAD outpatients would have better structure levels than GAD inpatients.

As one of our most important findings, the LASSO regression analysis showed that “self-worth conflict” and “total structure” were the most effective predictors, and two structure components “self-regulation” and “attachment capacity internal objects” were the second most effective predictors for distinguishing between the three groups. These results were consistent with the above-mentioned empirical evidence of MDD and GAD associated with lower self-esteem and insecure attachment. In addition, the results serve as evidence of distinct patterns of MDD/GAD-specific psychodynamic profiles, and therefore show the promise that the OPD could be used to assist the more precise diagnosis and interventions of depressive and anxiety patients in the clinics. Based on our data, various transdiagnostic and disorder-specific psychodynamic strategies about conflicts and structure could be designed for an evidence-based intervention for MDD and GAD. For instance, the psychodynamic therapy could consider expressive interventions of self-worth conflict for self-exploration to enhance self-esteem in GAD (6, 42, 43). Meanwhile, the psychodynamic therapy of MDD may focus more on structured and supportive interventions (6, 44, 45), such as promoting self-regulation ability, which was found as the lowest level among dimensions of substructures of MDD participants in our study. However, this only applies to patients on a group level, and each patient needs individualized treatment based on their precise OPD profiles. Moreover, the two substructures “self-regulation” and “attachment capacity internal objects” were found to be the most effective predictors when classifying groups. To a certain extent, our current results are consistent with the finding of Nowak and his team (37). They also found GAD outpatients tended to have impaired self-regulation. Additionally, the current profiles of conflicts in patients may be related to the characteristics of collective Chinese culture. The Chinese culture encourages emotional suppression and encourages people to “experience more other-focused emotions (e.g., sympathy and shame) rather than ego-focused emotions (e.g., anger, frustration, pride)” (46). Thus, the substructure levels of regulation of object-relationships and attachment capacity external objects could be enhanced in the Chinese culture, whereas, the substructure levels of “self-regulation” and “attachment capacity internal objects could be weakened among the Chinese.

Our research is not without limitations. First, the current study enrolled patients with no known comorbidity to aim for higher sample homogeneity, however ending up with a small sample size. Future studies should further expand the sample size to examine the reliability of psychodynamic profiles in China. Second, the effects of inpatient and outpatient subgroups and medicine on their psychodynamics among MDD and GAD patients in this study were not covered, inspiring future studies to further investigate. Third, it would be beneficial to include cultural characteristic assessments to explore the cultural influence on psychodynamic profiles for evidence-based and culture-specific psychotherapy approaches. Fourth, current results reveal psychodynamic patterns of MDD and GAD on a group-level, with limited ability to draw inferences on an individual-level. Together, OPD assessments compensate for the current clinical diagnosis of symptom-based diagnoses of MDD and GAD by revealing hidden psychodynamics and also provide a more economical approach to reveal subtypes of clinical populations in comparison to neuroimaging-based biotype models (47). Future studies should further expand the sample size, collect cultural characteristics, and investigate the impacts of inpatient/outpatient and medicine on psychodynamic profiles, and include self-assessed measures about psychodynamic profiles compensating other OPD assessments (48, 49).

## 5 Conclusions

In the current study, we used the OPD system to examine the multi-dimensional psychodynamics profiles of MDD, GAD patients, and health participants from China based on a small sample with relatively “pure” clinical symptoms. Results showed good inter-rater reliabilities of the OPD conflict and structure axes and revealed contrasts of conflict models and structure levels in MDD and GAD, promoting the understanding of psychodynamic profiles of MDD and GAD in China. The OPD system may serve as a complementary tool in addition to the classic symptom-based diagnosis system through revealing latent factors that indicate hidden causes of mental disorders and potentially guide treatment for therapists. In clinical practice, the understanding of psychodynamic profiles of a patient may serve as a useful indicator when carrying out clinical therapy intervention, facilitating personalized therapeutic interventions and more effective treatments.

## Data availability statement

The raw data supporting the conclusions of this article will be made available by the authors, without undue reservation.

## Ethics statement

The studies involving humans were approved by the ethics committee of Peking University Sixth Hospital (Institute of Mental Health) in China. The studies were conducted in accordance with the local legislation and institutional requirements. The participants provided their written informed consent to participate in this study.

## Author contributions

JX: Conceptualization, Data curation, Investigation, Methodology, Resources, Writing – original draft, Writing – review & editing. YW: Formal analysis, Methodology, Writing – review & editing. YP: Funding acquisition, Supervision, Writing – review & editing.
