# Supplementary material for: Psychodynamic profiles of major depressive disorder and generalized anxiety disorder in China
Source: Front Psychiatry. 2024 Jan 23;15:1312980. doi: 10.3389/fpsyt.2024.1312980 (PMC10844481; doi:10.3389/fpsyt.2024.1312980)
Supplement: Supplementary file 1 [file Table_1.docx]

Supplementary Materials for “Psychodynamic Formulations of Major Depressive Disorder and Generalized Anxiety Disorder in China”

Jia Xu, Yuxi Wang, Yujia Peng

# Supplementary Figures and Tables

## Supplementary Table

**Supplementary Table 1.** Psychodynamic profiles of groups.

| Variables |  | MDD group (A; n=42) | GAD group (B; n=32) | HC group (C; n=31) | *F* | Post-hoc *p_(_*_Bonferroni-corrected)_ | | |
| --- | --- | --- | --- | --- | --- | --- | --- | --- |
|  |  |  |  |  | A vs. B vs. C | A vs. B | A vs. C | B vs. C |
| Axis Ⅲ-conflict | | | | | | | | |
| Individuation vs. dependency | absent | 15(35.7) | 21(65.6) | 27(87.1) | 12.239*** | .028* | < .001*** | .102 |
|  | insignificant | 11(26.2) | 5(15.6) | 4(12.9) |  |  |  |  |
|  | significant | 6(14.3) | 2(6.2) | 0 |  |  |  |  |
|  | very significant | 10(23.8) | 4(12.5) | 0 |  |  |  |  |
| Submission vs. control | absent | 7(16.7) | 10(31.2) | 23(74.2) | 17.544*** | 1.000 | < .001*** | < .001*** |
|  | insignificant | 15(35.7) | 4(12.5) | 7(22.6) |  |  |  |  |
|  | significant | 13(31.0) | 11(34.4) | 1(3.2) |  |  |  |  |
|  | very significant | 7(16.7) | 7(21.9) | 0 |  |  |  |  |
| Need for care vs. autarky | absent | 11(26.2) | 13(40.6) | 25(80.6) | 11.236*** | 1.000 | < .001*** | .002** |
|  | insignificant | 15(35.7) | 7(21.9) | 4(12.9) |  |  |  |  |
|  | significant | 7(16.7) | 6(18.8) | 2(6.5) |  |  |  |  |
|  | very significant | 9(21.4) | 6(18.8) | 0 |  |  |  |  |
| Self-worth conflict | absent | 10(23.8) | 1(3.1) | 15(48.4) | 19.702*** | .050 | < .001*** | < .001*** |
|  | insignificant | 7(16.2) | 5(15.6) | 10(32.3) |  |  |  |  |
|  | significant | 12(28.6) | 12(37.5) | 6(19.4) |  |  |  |  |
|  | very significant | 13(31.0) | 14(42.8) | 0 |  |  |  |  |
| Guilt conflict | absent | 21(50.0) | 16(50.0) | 28(90.3) | 7.876*** | .931 | .001** | .023* |
|  | insignificant | 13(31.0) | 12(37.5) | 3(9.7) |  |  |  |  |
|  | significant | 3(7.1) | 4(12.5) | 0 |  |  |  |  |
|  | very significant | 5(11.9) | 0 | 0 |  |  |  |  |
| Oedipal conflict | absent | 21(50.0) | 19(59.4) | 24(77.4) | 4.434* | .702 | .011* | .287 |
|  | insignificant | 9(21.4) | 10(31.2) | 7(22.6) |  |  |  |  |
|  | significant | 6(14.3) | 1(3.1) | 0 |  |  |  |  |
|  | very significant | 3(7.1) | 2(6.2) | 0 |  |  |  |  |
|  | not ratable | 3(7.1) | 0 | 0 |  |  |  |  |
| Identity conflict | absent | 41(97.6) | 31(96.9) | 30(96.8) | 0.028 | 1.000 | 1.000 | 1.000 |
|  | insignificant | 1(2.4) | 1(3.1) | 1(3.2) |  |  |  |  |
| Main conflict | Individuation vs. dependency | 8(19.0) | 4(12.1) | 3(9.7) | - | - | - | - |
|  | Submission vs. control | 7(16.7) | 7(21.9) | 3(9.7) |  |  |  |  |
|  | Need for care vs. autarky | 10(23.8) | 6(18.8) | 5(16.1) |  |  |  |  |
|  | Self-worth conflict | 11(26.2) | 14(43.8) | 12(38.7) |  |  |  |  |
|  | Guilt conflict | 4(9.5) | 0 | 1(3.2) |  |  |  |  |
|  | Oedipal conflict | 2(4.8) | 1(3.1) | 3(9.7) |  |  |  |  |
|  | No | 0 | 0 | 4(12.9) |  |  |  |  |
| Followed by, in order of importance | Individuation vs. dependency | 5(11.9) | 1(3.1) | 2(6.5) | - | - | - | - |
|  | Submission vs. control | 17(40.5) | 11(34.4) | 3(9.7) |  |  |  |  |
|  | Need for care vs. autarky | 7(16.7) | 5(15.6) | 3(9.7) |  |  |  |  |
|  | Self-worth conflict | 9(21.4) | 9(28.1) | 3(9.7) |  |  |  |  |
|  | Guilt conflict | 3(7.1) | 4(12.5) | 1(3.2) |  |  |  |  |
|  | Oedipal conflict | 1(2.4) | 2(6.2) | 4(12.9) |  |  |  |  |
|  | Identity conflict | 0 | 0 | 1(3.2) |  |  |  |  |
|  | No | 0 | 0 | 14(45.2) |  |  |  |  |
| Mode of processing of main conflict | Predominantly active | 4(9.5) | 0 | 0 | 1.578 | .568 | .322 | 1.000 |
|  | Mixed but active | 15(35.7) | 19(59.4) | 18(66.7) |  |  |  |  |
|  | Mixed but passive | 14(33.3) | 12(37.5) | 8(29.6) |  |  |  |  |
|  | Predominantly passive | 9(21.4) | 1(3.1) | 1(3.7) |  |  |  |  |
|  | Not ratable | 0 | 1(3.1) | 0 |  |  |  |  |
| Mode of processing of followed conflict | Predominantly active | 1(2.4) | 0 | 0 | 5.560** | .027* | .018* | 1.000 |
|  | Mixed but active | 15(35.7) | 21(65.6) | 13(76.5) |  |  |  |  |
|  | Mixed but passive | 21(50.0) | 11(34.4) | 4(23.5) |  |  |  |  |
|  | Predominantly passive | 5(11.9) | 0 | 0 |  |  |  |  |
| Axis Ⅳ-structure | | | | | | | | |
| Self-perception | high | 0 | 0 | 28(90.3) | 120.694*** | < .001*** | < .001*** | < .001*** |
|  | 1.5 | 12(28.6) | 27(84.4) | 3(9.7) |  |  |  |  |
|  | moderate | 25(59.5) | 5(15.6) | 0 |  |  |  |  |
|  | 2.5 | 5(11.9) | 0 | 0 |  |  |  |  |
| Perception of the object | high | 0 | 0 | 27(87.1) | 103.538*** | < .001*** | < .001*** | < .001*** |
|  | 1.5 | 10(23.8) | 28(87.5) | 4(12.9) |  |  |  |  |
|  | moderate | 17(40.5) | 4(12.5) | 0 |  |  |  |  |
|  | 2.5 | 13(31.0) | 0 | 0 |  |  |  |  |
|  | low | 2(4.8) | 0 | 0 |  |  |  |  |
| Self-regulation | high | 0 | 0 | 23(74.2) | 147.417*** | < .001*** | < .001*** | < .001*** |
|  | 1.5 | 0 | 10(31.2) | 8(25.8) |  |  |  |  |
|  | moderate | 23(54.8) | 22(68.8) | 0 |  |  |  |  |
|  | 2.5 | 14(33.3) | 0 | 0 |  |  |  |  |
|  | low | 5(11.9) | 0 | 0 |  |  |  |  |
| Regulation of the object relation | high | 0 | 0 | 20(64.5) | 89.122*** | < .001*** | < .001*** | < .001*** |
|  | 1.5 | 3(7.1) | 14(43.8) | 11(35.5) |  |  |  |  |
|  | moderate | 24(57.1) | 17(53.1) | 0 |  |  |  |  |
|  | 2.5 | 10(23.8) | 1(3.1) | 0 |  |  |  |  |
|  | low | 5(11.9) | 0 | 0 |  |  |  |  |
| Internal communication | high | 0 | 0 | 25(80.6) | 95.283*** | < .001*** | < .001*** | < .001*** |
|  | 1.5 | 6(14.3) | 10(31.2) | 5(16.1) |  |  |  |  |
|  | moderate | 17(40.5) | 21(65.6) | 1(3.2) |  |  |  |  |
|  | 2.5 | 15(35.7) | 1(3.1) | 0 |  |  |  |  |
|  | low | 4(9.5) | 0 | 0 |  |  |  |  |
| Communication with the outside world | high | 0 | 0 | 23(74.2) | 87.420*** | < .001*** | < .001*** | < .001*** |
|  | 1.5 | 9(21.4) | 26(81.2) | 8(25.8) |  |  |  |  |
|  | moderate | 18(42.9) | 6(18.8) | 0 |  |  |  |  |
|  | 2.5 | 13(31.0) | 0 | 0 |  |  |  |  |
|  | low | 2(4.8) | 0 | 0 |  |  |  |  |
| Internal objects | high | 0 | 0 | 24(77.4) | 137.729*** | < .001*** | < .001*** | < .001*** |
|  | 1.5 | 1(2.4) | 14(43.8) | 7(22.6) |  |  |  |  |
|  | moderate | 26(61.9) | 18(56.2) | 0 |  |  |  |  |
|  | 2.5 | 12(28.6) | 0 | 0 |  |  |  |  |
|  | low | 3(7.1) | 0 | 0 |  |  |  |  |
| External objects | high | 0 | 0 | 27(87.1) | 100.318*** | < .001*** | < .001*** | < .001*** |
|  | 1.5 | 8(19.0) | 22(68.8) | 4(12.9) |  |  |  |  |
|  | moderate | 20(47.6) | 10(31.2) | 0 |  |  |  |  |
|  | 2.5 | 11(26.2) | 0 | 0 |  |  |  |  |
|  | low | 3(7.1) | 0 | 0 |  |  |  |  |
| Total structure | high | 0 | 0 | 28(90.3) | 202.512*** | < .001*** | < .001*** | < .001*** |
|  | 1.5 | 1(2.4) | 22(68.8) | 3(9.7) |  |  |  |  |
|  | moderate | 25(59.5) | 10(31.2) | 0 |  |  |  |  |
|  | 2.5 | 15(35.7) | 0 | 0 |  |  |  |  |
|  | low | 1(2.4) | 0 | 0 |  |  |  |  |

* *p* < .05 ** *p* < .01 *** *p* < .001. MDD = major depressive disorder, GAD = generalized anxiety disorder, HC = healthy controls.

**Supplementary Table 2.** The predictor effectiveness for the LASSO regression.

| Predictors | Effectiveness ^c^ | HC | | GAD | | MDD | |
| --- | --- | --- | --- | --- | --- | --- | --- |
|  |  | Estimate | CI [min, max] | Estimate | CI [min, max] | Estimate | CI [min, max] |
| Conflict of self-worth ^a^ | 100% | -0.25 | [-1.01, 0.00] | 0.35 | [0.00, 1.96] | -0.10 | [-1.22, 0.07] |
| Structure total ^b^ | 99% | -1.40 | [-3.81, 0.00] | -0.38 | [-2.84, 0.02] | 1.78 | [0.00, 5.50] |
| Internal objects ^b^ | 98% | -1.08 | [-5.40, 0.00] | -0.03 | [-1.71, 0.37] | 1.11 | [0.00, 6.97] |
| Self-regulation ^b^ | 96% | -0.98 | [-6.57, 0.00] | -0.10 | [-4.35, 0.28] | 1.08 | [0.00, 10.92] |
| Object perception ^b^ | 75% | -0.79 | [-7.95, 0.00] | -0.44 | [-8.97, 0.00] | 1.23 | [0.00, 16.92] |
| Self-perception ^b^ | 58% | -0.85 | [-4.88, 0.00] | 0.21 | [-1.38, 1.95] | 0.64 | [-0.44, 5.96] |
| Internal communication ^b^ | 54% | -0.64 | [-2.87, 0.00] | 0.67 | [0.00, 4.15] | -0.04 | [-2.02, 0.80] |
| Submission vs. control ^b^ | 40% | -0.14 | [-0.68, 0.00] | 0.02 | [-0.37, 0.73] | 0.12 | [-0.32, 1.05] |
| Second conflict ^a^ | 39% | 0.01 | [-0.09, 0.32] | 0.15 | [-0.03, 1.08] | -0.16 | [-1.22, 0.13] |
| Need for care vs. autarky ^a^ | 36% | -0.27 | [-0.78, 0.00] | 0.21 | [0.00, 0.78] | 0.06 | [-0.18, 0.58] |
| Main conflict ^a^ | 36% | 0.04 | [-0.07, 0.26] | -0.39 | [-1.83, 0.00] | 0.35 | [0.00, 1.83] |
| External objects ^b^ | 34% | -0.50 | [-1.82, 0.00] | -0.09 | [-1.03, 0.06] | 0.59 | [-0.01, 2.85] |
| Oedipal conflict ^a^ | 30% | -0.18 | [-0.44, 0.00] | 0.57 | [0.00, 1.95] | -0.39 | [-1.65, 0.00] |
| Guilt conflict ^a^ | 25% | -0.18 | [-0.67, 0.00] | 0.25 | [-0.13, 1.10] | -0.07 | [-0.79, 0.80] |
| Individuation vs. dependency ^a^ | 20% | -0.11 | [-0.26, 0.00] | -0.16 | [-0.52, 0.07] | 0.27 | [-0.03, 0.66] |
| Communication with the outside world ^b^ | 17% | -0.25 | [-1.25, 0.00] | -0.04 | [-0.71, 1.88] | 0.29 | [-1.68, 1.96] |
| Regulation of the object relation ^b^ | 15% | -0.27 | [-0.91, 0.00] | 0.55 | [0.00, 2.41] | -0.27 | [-1.50, 0.01] |
| Response mode to main conflict ^a^ | 14% | -0.10 | [-0.38, 0.00] | 0.02 | [-0.21, 0.80] | 0.09 | [-0.42, 0.54] |
| Response mode to second conflict ^a^ | 9% | -0.05 | [-0.62, 0.62] | -0.28 | [-2.23, 0.59] | 0.32 | [-1.21, 2.85] |
| Identity conflict ^a^ | 4% | -0.27 | [-1.01, 0.03] | 0.34 | [-0.04, 1.30] | -0.07 | [-0.29, 0.01] |

^a^ Predictors belong to Axis III conflicts; ^b^ Predictors belong to Axis IV structures; ^c^ Effectiveness indicates the percentage of each predictor being selected across 100 iterations of model fitting; MDD = major depressive disorder, GAD = generalized anxiety disorder, HC = healthy controls.
